# Une observation d'un goitre sur dysgénésie thyroïdienne pré-sternale

**DOI:** 10.11604/pamj.2015.21.117.6919

**Published:** 2015-06-12

**Authors:** Herinirina Nicolas Fanantenana, Rajaonarison Ny Ony Narindra Lova Hasina, Rafanomezantsoa Hery, Rakotoarisoa Andriamihaja Jean Claude, Ahmad Ahmad

**Affiliations:** 1 Service Imagerie Médicale, Centre Hospitalier Universitaire d'Antsiranana, Madagascar; 2 Service Imagerie Médicale, Centre Hospitalier Universitaire Joseph Ravoahangy Andrianavalona, Antananarivo, Madagascar; 3 Service de Chirurgie Thoracique, Centre Hospitalier Universitaire Joseph Ravoahangy Andrianavalona, Antananarivo, Madagascar

**Keywords:** Hétérotopie thyroïdienne, pré-sternale, histologie, chirurgie, thyroid enlargement, pre-sternale, histology, surgery

## Abstract

L'hétérotopie thyroïdienne est une localisation anormale de tissus thyroïdiens normaux coexistant avec un organe normal et de localisation normale. Elle se distingue de l'ectopie thyroïdienne et d'une métastase du cancer de la thyroïde. Nous rapportons un cas d'hétérotopie thyroïdienne pré-sternale chez une femme de 65 ans afin de discuter le mécanisme de la migration de la thyroïde, le problème diagnostique et thérapeutique posé par cette topographie exceptionnelle. L'origine thyroïdienne de la masse est confirmée par l'histologie. Dans les lieux isolés, la chirurgie d'exérèse reste le seul moyen pour avoir le diagnostic et pour traiter les patients.

## Introduction

Les dysgénésies thyroïdiennes regroupent l'ensemble des anomalies du développement de la glande thyroïde, et incluent, les hypoplasies, les ectopies, anomalies de la migration (60 à 65% des cas) et les athyréoses ou absences complètes de glande (30 à 35%) [1]. L'ectopie thyroïdienne est caractérisée par l'absence de la glande en regard du 2^ème^ au 4^ème^ cartilage trachéal [2]. L'ectopie thyroïdienne haute est la plus fréquente, très souvent linguale, se rencontre dans 90% des cas [3]. L'ectopie basse médiastinale ou thyroïde endothoracique se voit plus fréquemment chez les femmes. D'autres anomalies de siège peuvent se voir au cours de la migration thyroïdienne comme la thyroïde pré-laryngée et intra-trachéale. L'hétérotopie thyroïdienne est une anomalie congénitale de la situation d'un tissu thyroïdien coexistant avec une thyroïde normale et en position normale. Nous rapportons le cas d'une femme ayant présenté une masse pré-sternale diagnostiquée comme un goitre thyroïdien hétéronodulaire.

## Patient et observation

**Figure 1:**
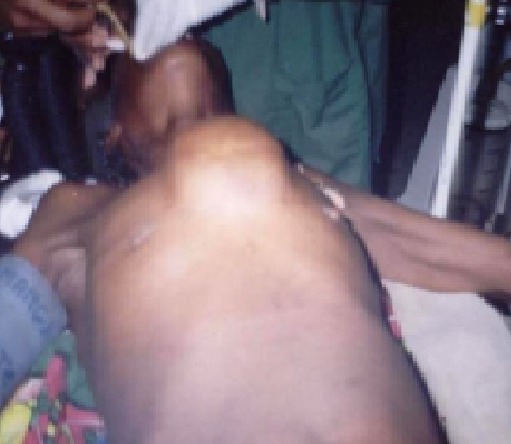
Volumineuse masse pré-sternale

**Figure 2:**
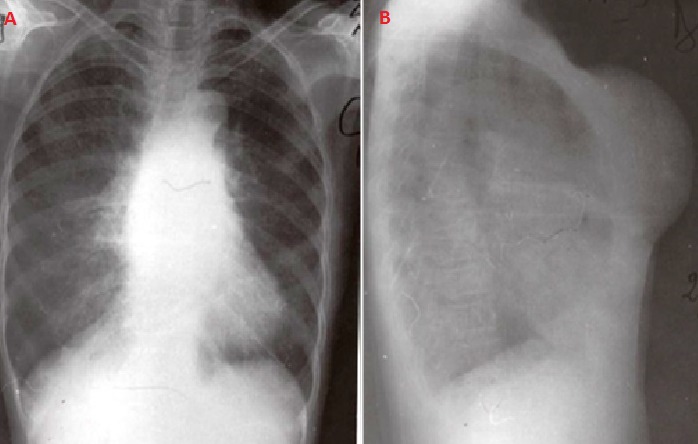
Radiographie du thorax en incidence de face (A) et profil (B) montrant une opacité pré-sternale avec lyse osseuse sternale

**Figure 3:**
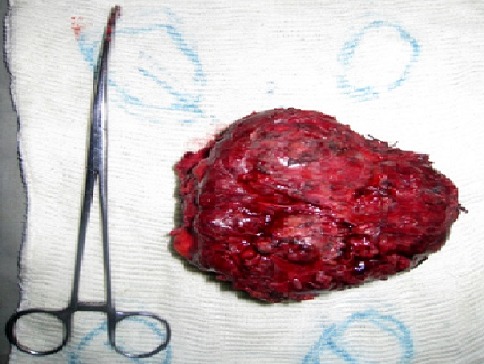
Aspect macroscopique de la pièce opératoire

**Figure 4:**
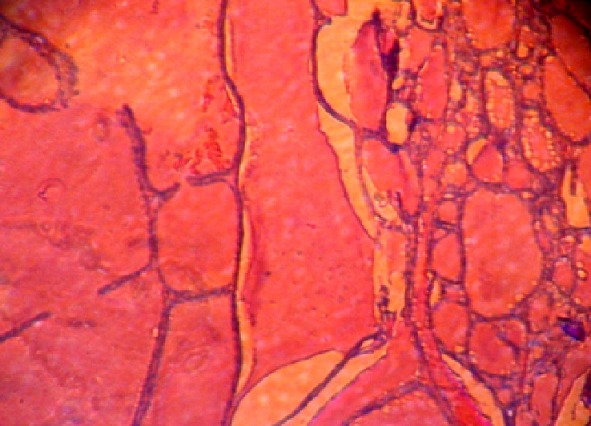
Aspect microscopique d'un goitre hétéronodulaire avec thyréocytes normaux

Une femme de 60 ans, cultivatrice, mère de 7 enfants et vivant seule a été adressée dans le centre hospitalier de district de Moramanga, Madagascar le mois de décembre 2005, pour une tuméfaction pré-sternale indolore mais gênant les activités habituelles. A l'anamnèse, il s'agissait d'une tumeur récidivante ayant déjà bénéficiée d'une exérèse en 1998 et dont la pièce opératoire, n'a pas été envoyée pour examen anatomo-pathologique. La patiente ne présentait aucun antécédent particulier. Avant son admission, elle a constaté que le volume de la tumeur a été triplé durant les six derniers mois empêchant de réaliser ses activités habituelles. A l'examen, la patiente était apyrétique, les mains chaudes et moites et elle présentait une hypersudation. Elle pesait 57,5 kg pour une taille de 158 cm. La tension artérielle était de 160/80 mmHg, la pulsation à 102/minutes et la fréquence respiratoire 22/minutes. Une tuméfaction médio-thoracique de 18 x 14 cm était visible et palpable (Figure 1). Cette lésion était adhérente au plan profond mais mobile par rapport au plan cutané. La masse était indolore, de consistance ferme à la palpation mais sa compression provoquait une douleur sternale. Il n'y avait pas de ganglions superficiels palpables. L'examen électrocardiographique ne montrait qu'une tachycardie sinusale. Le bilan biologique montrait: un nombre de globule rouge à 3 950 000 / mm3, un nombre de globule blanc à 7800/mm^3^, un taux de polynucléaire neutrophile à 55%, un taux de polynucléaire éosinophile à 44%, un taux de lymphocyte de 41%, un temps de saignement à 2'18’‘, un temps de coagulation à 4'45’‘, un bilan rénal composé de: Azotémie à 0,4g /l; créatinémie à 0,6 mg /dl, une vitesse de sédimentation des hématies normales, une glycémie à 1,30g/l. Le bilan morphologique tel que la radiographie thoracique standard (Figure 2) prise en incidence de face ne montrait aucune anomalie à part une opacité homogène en projection sur l'ombre médiastinale, en rapport avec la masse. En incidence de profil, l'opacité était de localisation pariétale pré-sternale et s'accompagnait d'une érosion de la face antérieure du sternum. L’échographie de la masse et le scanner thoracique n'ont pas pu être effectuées, ainsi que l'exploration fonctionnelle respiratoire; faute de plateau technique. Une intervention chirurgicale était réalisée. L'exérèse de la masse était laborieuse sur le plan profond car la lésion était très adhérente au niveau du sternum. Une partie du périoste et de l'os spongieux ont été enlevées. Cette ablation nécessitait une hémostase minutieuse car la tumeur était hypervascularisée. La tumeur était de consistance ferme, plus ou moins lisse, ovoïde et encapsulée surtout dans sa partie antérieure. À l'incision, la tumeur était cérébroïde et l'ablation en bloc était possible (Figure 3). La pièce opératoire était ensuite envoyée au laboratoire pour examen histologique après fixation au formol de 10%. La suite opératoire immédiate était simple en dehors de la douleur. La tension artérielle était stable (130/85 mmHg). Les paramètres biologiques ne changeaient pas par rapport au bilan préopératoire. La patiente était sortie au dixième jour de son intervention. Le résultat de l'examen anatomo-pathologique révélait un goitre thyroïdien hétéronodulaire (Figure 4), sans signes de malignité. La patiente est perdue de vue et ne se montrait plus pour la surveillance postopératoire. Aucun examen pour vérifier la présence de la glande thyroïde normale n'est effectuée tel que l’échographie ou la scintigraphie.

## Discussion

L'hétérotopie c'est une localisation anormale de tissus normaux coexistant avec un organe normal et de localisation normale qu'il faut différencier avec la métastase de cancer. L'ectopie thyroïdienne est une anomalie congénitale responsable de la topographie anormale de la glande thyroïde. Elle est caractérisée par l'absence de la glande en regard du 2^ème^ au 4^ème^ cartilage trachéal [2]. L'ectopie et l'hétérotopie sont dues à des anomalies de la maturation des tissus thyroïdiens. La fréquence d'une ectopie de la glande thyroïde varie entre 7 à 10%. La thyroïde ectopique haute (linguale) se rencontre dans 90% des cas. L'ectopie basse (médiastinale: retro-sternale et endothoracique) s'objective dans 10% des cas [3]. La situation pré-sternale d'une thyroïde sans signe de malignité, comme le cas de cette femme, reste exceptionnelle. Les cas rapportés sont des goitres malins par extension locorégionale de la tumeur [4–6]. L’âge de découverte de l'ectopie ou de l'hétérotopie de la glande thyroïde est variable et dépend de la manifestation clinique. D'après Cérulus, les goitres intrathoraciques se voient surtout chez les femmes de plus de 45 ans [7]. Aucune race n'est épargnée de cette pathologie mais la race jaune serait plus touchée par la dysgénésie thyroïdienne contrairement à la race noire qui est moins affectée [8]. Plusieurs gênes ont été impliquées dans les dysgénésies thyroïdiennes. Des mutations de gène Pax-8 ont été trouvées chez 05 sujets atteints d'hypothyroïdie congénitale: un cas d'ectopie, un cas d'hypoplasie et un cas familial d'hypoplasie de la glande thyroïde (une mère et ses deux enfants) [9]. Pour notre patiente, le mécanisme de cette migration des tissus thyroïdiens est encore non élucidé. Par contre, le mécanisme de formation des tissus ou organes aberrants peut résulter d'un blocage au cours de la migration ou par division d'une ébauche embryonnaire et/ou détournement d'un fragment de l’ébauche. Le diagnostic de la dysgénésie thyroïdienne dépend de sa localisation, de son phénotype (athyréose, hypoplasie, ectopie) et son expression fonctionnelle (hypothyroïdie, hyperthyroïdie, euthyroïdie).

La situation anormale des tissus thyroïdiens dans notre observation et l'insuffisance des examens complémentaires posent la discussion entre l'hétérotopie thyroïdienne et l'ectopie thyroïdienne. Pour l'ectopie haute le diagnostic peut se faire à la naissance lors du dépistage biologique systématique de l'hypothyroïdie. Les ectopies linguales de petite taille peuvent ne pas être détectées [10]. Chez les enfants plus âgés, l'attention peut être attirée par un nanisme inexpliqué voire un retard psychomoteur [11]. Ces différents signes n’étaient pas observés chez notre patiente. Chez l'adulte, la clinique évoque surtout une hypothyroïdie franche. Rarement, elle se traduit soit par un phénomène compressif: toux, dysphagie, dysphonie, dyspnée, soit par une complication de l'hypothyroïdie en l'absence de traitement; il s'agit le plus souvent de femmes jeunes (30% au moment de la puberté, 55% entre 18 et 40 ans) [11–13]. A l'examen clinique, l'absence de la thyroïde cervicale et l'existence d'une masse linguale postérieure palpable évoque le diagnostic. La biologie montre le plus souvent une TSH très élevée. Le dosage des anticorps antithyroïdiens est habituellement normal. L’échographie montre une loge thyroïdienne vide et permet parfois de localisée la glande ectopique. La scintigraphie thyroïdienne au 99mTechnétium ou à l'iode 123 pose le diagnostic et permet de confirmer cette hypothèse d'hypothyroïdie. Elle montre l'hyperactivité au niveau de la base de la langue sans zone d'activité dans la région cervicale antérieure [14]. La tomodensitométrie (TDM) apprécie la densité de la masse, ses limites, son siège et ses rapports avec les axes vasculaires. L'IRM, en séquences écho de spin T1 (sans et avec injection de gadolinium) et T2 dans au moins deux plans perpendiculaires, permet d'explorer la masse dans son grand axe, d'identifier un éventuel contingent vasculaire tumoral, de détecter les extensions péri-neurales ainsi que de localiser avec précision les rapports aux axes vasculaires. Notons que pour les thyroïdes ectopiques, le diagnostic est surtout radiologique [15, 16]. Pour l'ectopie basse, il est à différencier des goitres intra-thoraciques secondaires au développement d'une glande initialement en position normale (goitre plongeant). La découverte en est souvent fortuite par la radiographie du thorax. Ces glandes ectopiques, même de petites tailles peuvent entraîner une compression trachéale, ‘sophagienne si elles sont situées à hauteur du défilé thoracique. Orientée par la radiographie du thorax, la scintigraphie, permet d'objectiver du tissu thyroïdien fonctionnel dans le médiastin. Le point capital est de la réaliser avant le CT-Scan avec injection de produit de contraste qui inhiberait la captation du traceur [17]. Plus rarement, l'absence de captation peut aussi correspondre à du tissu thyroïdien non fonctionnel. La barrière osseuse thoracique rend l’échographie peu performante. L'hyperthyroïdie est rarement observée au cours d'une ectopie thyroïdienne.

L'hétérotopie thyroïdienne est, par contre, un tissu simple ou composé d'origine thyroïdienne qui peut s'organiser pour former un organe entier dans n'importe quel endroit du corps où normalement il ne doit pas s'y situé. Sur le plan fonctionnel, la glande hétérotopique peut sécréter des hormones comme une vraie glande thyroïde [18]. Cette hétérotopie est d’évolution latente, ou se présente par un aspect tumoral [19]. Elle est souvent de découverte fortuite lors d'un examen scintigraphique d'une glande thyroïde présentant des troubles fonctionnels (hyperthyroïdie ou hypothyroïdie) [20]. Si la glande se développe vers l'extérieur, c'est-à-dire au niveau de la paroi, elle se présente comme une simple tuméfaction. Douloureuse ou non, le diagnostic au cours d'une scintigraphie est difficile devant un tissu aberrant hypofixant ou non fonctionnel. L’échographie permet de distinguer l'aspect tissulaire ou kystique de la glande. Seul, l'examen anatomopathologique d'une biopsie ou d'une pièce opératoire affirme l'origine thyroïdienne de la tuméfaction et permet d’éliminer une métastase cancéreuse d'origine thyroïdienne. Les lésions intra-thoraciques ou intra-abdominales seront découverte fortuitement lors d'une scopie, laparotomie, thoracotomie ou à l'autopsie [21]; ou sous la forme d'une masse lors de l'imagerie: échographie, scannographie, IRM [22]. Notre patiente n'a présenté aucun signe fonctionnel, sauf une dyspnée légère au cours d'une montée sur une pente. L'examen physique a révélé une tachycardie, une légère tachypnée, une hypersudation, une hypertension artérielle systolique et une masse pré-sternale volumineuse qui peuvent être en faveur d'une hyperthyroïdie. La glande thyroïde normale n'est pas examinée car il était difficile de rattacher la tuméfaction pré-sternale avec la glande thyroïde, avant le résultat de l'anatomo-pathologique, pour confirmer l'hétérotopie. De plus, la patiente était ensuite perdue de vue. De même, aucun examen paraclinique spécifique pour la thyroïde n'a été réalisé (imagerie et biologie: T3, T4, TSH). Ces bilans pourraient orienter le diagnostic et guider une biopsie chirurgicale, indispensable et préalable à toute prise en charge thérapeutique ultérieur. L'examen histologique de la pièce d'exérèse a permis de mettre en évidence l'origine thyroïdienne avec un aspect de goitre hétéronodulaire sans signe de malignité. Devant l'absence de problème particulier de son développement psychomoteur pendant toute sa vie, le développement à l’âge adulte de la masse pré-sternale et au vu du résultat anatomopathologique, nous retenons le diagnostic de hétérotopie thyroïdienne. La latence clinique est environ 55 ans pour notre cas. Une longue période de latence est en faveur d'une hétérotopie [19], comme le diverticule de Meckel et l'endométriose. L’évolution de la masse est d'allure bénigne avec absence d'altération de l’état générale mais survenue de récidive tumorale après dix ans de la première chirurgie exérèse qui est probablement incomplète. De plus, il n'y avait pas d'adénopathies superficielles.

Vu le manque des examens complémentaires, la prise en charge thérapeutique de la patiente n'est qu'univoque. D'ailleurs, la conduite à tenir face à une tumeur de la paroi thoracique reste l'ablation chirurgicale en monobloc [23]. Comme pour toutes les lésions tumorales de la paroi thoracique, il est important de réaliser un bilan d'extirpabilité de la tumeur. Pour cela, l'imagerie en coupe (échographie, scanner, IRM) tient son importance capitale [11, 24]. Dans les lieux isolés, comme notre cas, où les examens paracliniques sont inaccessibles, la chirurgie d'exérèse reste le seul moyen pour avoir le diagnostic et pour traiter les patients. Les bilans hormonaux thyroïdiens restent utiles pour savoir l'activité de la glande thyroïde avant et surtout après la chirurgie pour une conduite adéquate.

## Conclusion

L'hétérotopie thyroïdienne pré-sternale est exceptionnelle. Le cas rapporté pose un problème diagnostic devant une tumeur de la paroi thoracique. Malgré leur diversité anatomo-clinique, les lésions de la paroi thoracique ont des caractères communs: douleur et/ou tuméfaction, ostéolyse ou déformation osseuse. Ces manifestations cliniques ne sont pas spécifiques, des investigations paracliniques tel que l'imagerie sont nécessaires. La scintigraphie et l’échographie sont très performantes dans le diagnostic positif; la tomodensitométrie et l'IRM jouent un rôle important dans le bilan pré-thérapeutique. Le diagnostic formel est histologique après biopsie ou exérèse de la lésion.
